# Supplementary material for: The Gut Bacteria Dysbiosis Contributes to Chronic Graft-Versus-Host Disease Associated With a Treg/Th1 Ratio Imbalance
Source: Front Microbiol. 2022 Sep 8;13:813576. doi: 10.3389/fmicb.2022.813576 (PMC9493085; doi:10.3389/fmicb.2022.813576)

_MD5: 0.954595572644 (of 1.0) from 19 categories displayed, excluding 0.0454044273557 from 80 categories ('All Other Categories')

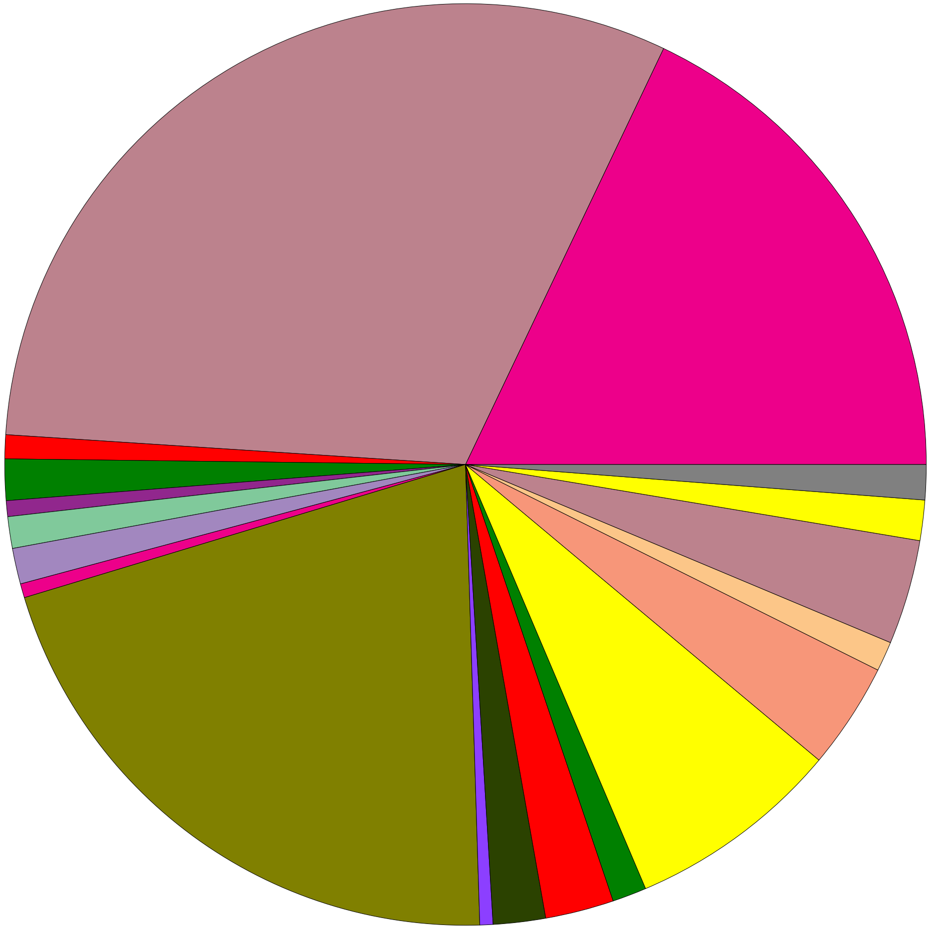

Supplement: Supplementary file 1 [file Data_Sheet_1.zip › P101SC18090073-01-B1-3-4_result/05.WebShow/taxa_charts_html/charts/L6gK7HEgx5mS5s1CUO2un0N6NlXbz7.pdf]
